# Carbenoxolone-mediated cytotoxicity inhibits *Vaccinia virus* replication in a human keratinocyte cell line

**DOI:** 10.1038/s41598-018-34732-w

**Published:** 2018-11-16

**Authors:** Ismar R. Haga, Jennifer L. Simpson, Philippa C. Hawes, Philippa M. Beard

**Affiliations:** 1 The Roslin Institute, University of Edinburgh, Easter Bush, Midlothian, EH25 9RG United Kingdom; 2 The Pirbright Institute, Ash Road, Pirbright, Woking, Surrey, GU24 0NF United Kingdom

## Abstract

The re-emergence of poxviral zoonotic infections and the threat of bioterrorism emphasise the demand for effective antipoxvirus therapies. Here, we show that carbenoxolone, a pharmacological inhibitor of gap junction function and a compound widely used in cell culture, is capable of hindering the replication of *Vaccinia virus*, the prototypical poxvirus, in a gap junction-independent manner in a human keratinocyte cell line. Viral protein synthesis occurs in the presence of carbenoxolone but infectious virion formation is minimal, indicating that carbenoxolone blocks viral morphogenesis. Initial viability tests suggested that carbenoxolone was not toxic to cells. However, electron microscopic analysis of carbenoxolone treated cells revealed that it alters the cellular endomembrane system. This widespread ultrastructural damage prevents *Vaccinia virus* virion assembly. These results strengthen the need for thorough characterisation of the effects of antiviral compounds on the cellular ultrastructure.

## Introduction

*Vaccinia virus* (VACV) is the prototypical member of the *Poxviridae*, a family of large DNA viruses that replicate entirely in the cytoplasm of infected cells. VACV is widely used as a biological tool and expression vector, as well as an oncolytic therapy. Recently, a number of zoonotic infections with poxviruses such as *Monkeypox virus* and *Cowpox virus*^1^ in addition to VACV^2^ have been reported, highlighting the need for safe and effective antipoxvirus treatments. We investigated a panel of novel inhibitors of VACV replication (data not shown) and identified carbenoxolone (CBX) as a potent inhibitor of virus growth.

CBX is a semi synthetic compound derived from glycyrrhizic acid (GZA), found in the root of liquorice (*Glycyrrhiza glabra*). It has been shown to possess anti-inflammatory properties^3–5^ and was previously used in the treatment of peptic ulcers with relatively uncommon side effects^3^. It has also been shown to protect against LPS-induced PKR activation^4^ and ischemic injury^6^ and regulate the expression of heat shock proteins^7^. CBX has been widely used in cell culture experiments as an uncoupling agent for gap junctions^8^. Gap junctions are clusters of intercellular channels, composed of membrane proteins named connexins, which permit communication between adjacent cells^9,10^. CBX has been reported to reduce VACV growth in L929 cells^11^, however its mechanism of action on VACV replication has not been determined.

As poxviruses are well-known to replicate and cause pathology in keratinocytes, we opted to investigate the effect of CBX in VACV replication in a human keratinocyte cell line.

## Results and Discussion

**Figure 1:**
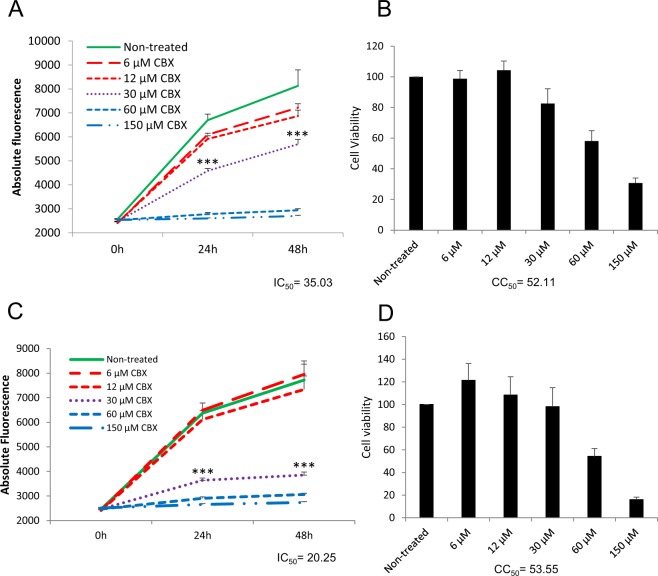
Carbenoxolone inhibits VACV replication in a gap junction-independent manner. Cells ((**A**) HaCaT and (**C**) N2a) were untreated or treated with different concentrations of CBX and then infected with VACV-A5L-EGFP (MOI = 0.1). Virus levels were determined by fluorescence. Results of untreated cells and cells treated with 30 µM CBX were analysed by t test, *** denotes p < 0.05. Cell viability ((**B**) HaCaT and (**D**) N2a) was defined by CellTiter-Blue ® assay. Non-treated cells were designated as 100% viability. IC_50_ and CC_50_ values (48 h p.i.) are noted. Experiments are representative of two independent replicates performed in triplicate. Error bars = standard deviation.

HaCaT cells, a spontaneously immortalised human keratinocyte line^12^, were pre-treated for 1 h with different concentrations of CBX or carrier prior to infection with VACV-A5L-EGFP, a VACV construct in which the core A5 protein has been tagged with EGFP^13^, allowing estimation of virus growth on the basis of fluorescence levels^14^. Subsequent steps in cells pre-treated with CBX were also carried out in the presence of the compound. VACV infection was carried out at 0.1 pfu/cell (MOI = 0.1) and fluorescence levels were measured at 0, 24 and 48 h p.i. We observed that CBX inhibited VACV replication in a dose-dependent manner (Fig. 1A). Toxicity levels in cells treated with CBX were compared to the carrier control at 48 h p.i. using Cell-Titer Blue ® (Promega) which measures toxicity through the ability of viable cells to metabolise resazurin into fluorescent resorufin. No evidence of toxicity was identified up to 30 µM.

CBX has been broadly used as gap junction inhibitor^3,8,15^. To define whether gap junctions have a role in the CBX-induced inhibition of VACV replication, we used the murine neuroblastoma cell line N2a, which has been reported to lack known endogenous connexins (the components of gap junctions^8^). CBX was able to efficiently inhibit VACV replication in these connexin-deficient cells (Fig. 1C), indicating that the impact of CBX on VACV replication is connexin- and, therefore, gap junction-independent. CBX does not cause significant cell death in N2a cells as detected by Cell-Titer Blue (Fig. 1D) up to concentrations of 30 µM, similar to the HaCaT cells (Fig. 1B), and we chose this concentration of CBX for the remaining experiments.

Apart from its gap junction blocking properties, CBX has been shown to increase production of protein phosphatase 2 A (PP2A) in microglia^3^. This is relevant to the current study because PP2A is involved in VACV replication through its role in dephosphorylating the barrier-to-autointegration factor (BAF) protein during virus infection. BAF binds VACV DNA and hinders its replication, therefore working as an anti-viral factor. VACV possesses numerous ways of counteracting intracellular defenses and it thwarts BAF by the action of the B1 kinase, which phosphorylates BAF, reducing its DNA binding activity. PP2A opposes this activity, dephosphorylating BAF during VACV infection and consequently enhancing BAF binding to viral DNA^16^. Thus, PP2A activity promotes BAF’s anti-viral role in the context of VACV infection.

**Figure 2:**
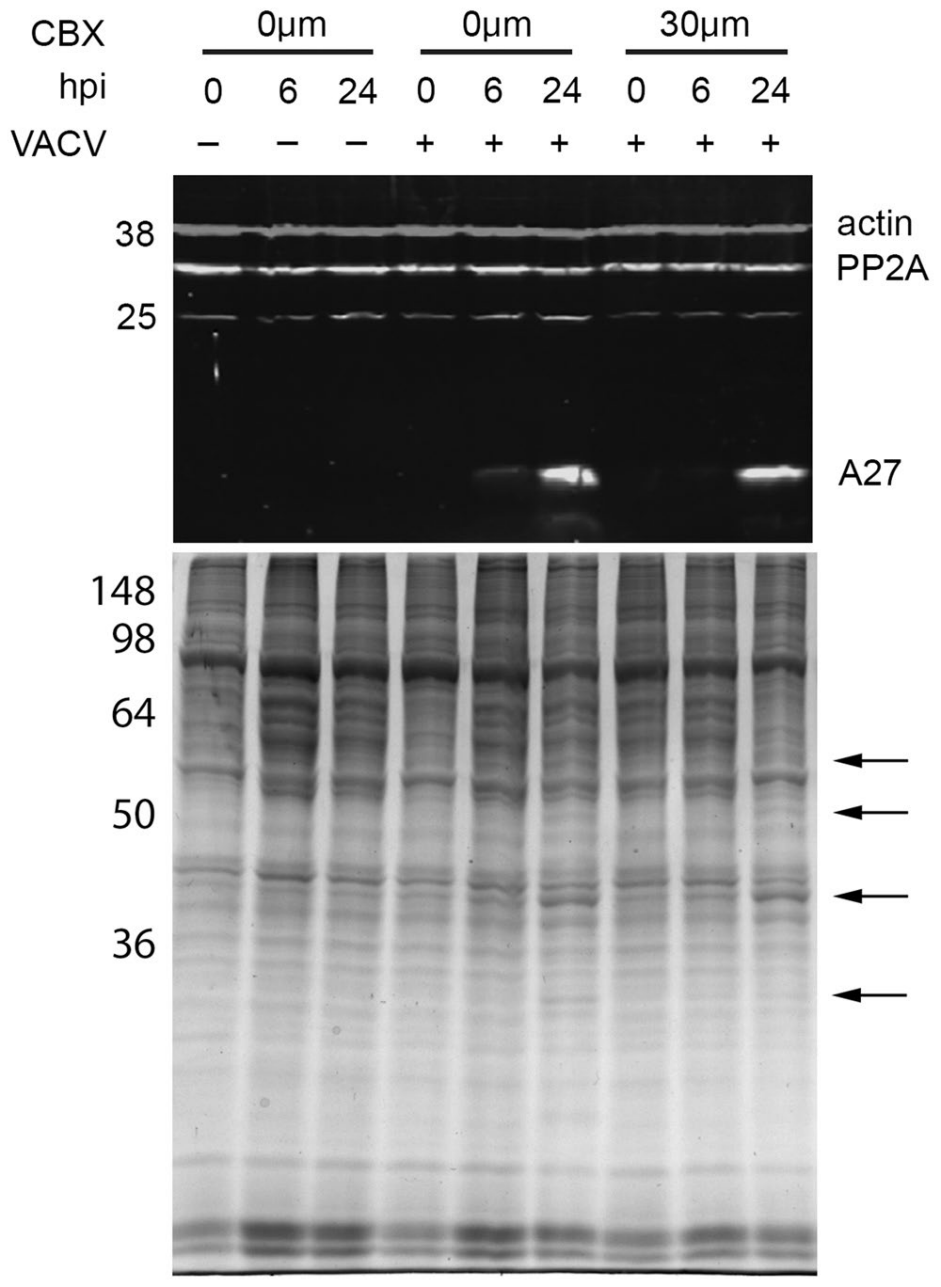
Carbenoxolone treatment does not alter the expression of PP2A or VACV proteins. Top panel: HaCaT cells were mock-treated or treated with 30 µM CBX and then infected with VACV (MOI = 5). Whole cell lysates were collected at the indicated time points and analysed by immunoblotting with the indicated antibodies. Bottom panel: HaCaT cells were mock-treated or treated with 30 µM CBX and then infected with VACV (MOI = 5). Whole cell lysates were collected at the indicated time points, 5 µg of protein were fractionated by SDS-PAGE and visualised by SimplyBlue™ staining. Arrows indicate VACV-encoded proteins. Numbers: position of molecular weight markers (in kDa).

As CBX has been shown to upregulate PP2A and PP2A upregulation can lead to an inhibition of VACV DNA replication, we hypothesised that the inhibitory effect of CBX in VACV replication could be linked to an upregulation of PP2A in human keratinocytes. In order to investigate this, HaCaT cells were pre-treated with 30 µM CBX or left untreated for 1 h and either infected with VACV at an MOI of 5 or mock infected. Whole cell lysates were collected at 0, 6 and 24 h p.i. and used for Western blot analysis. No change in the expression of PP2A was observed in response to either CBX treatment or VACV infection of HaCaT cells (Fig. 2, top panel). These results show that PP2A is not upregulated by CBX in HaCaT cells. It is unlikely, therefore, that PP2A hindering of VACV replication is the mechanism by which CBX induces inhibition of VACV growth.

We next sought to determine the influence of CBX in viral protein production. The cell lysates described above were blotted against an antibody raised against A27, a late VACV protein^17^. There was a strong A27 band detected in the lane containing lysate from cells treated with CBX (Fig. 2, top panel), suggesting that late viral protein production and hence viral DNA replication take place in the presence of CBX (as VACV late proteins are only produced after this process occurs^17^). We further analysed the effect of CBX in VACV protein production by SimplyBlue™ staining. We observed the presence of multiple virus-encoded proteins in cells that had been treated with CBX (Fig. 2, bottom panel, arrows), indicating that CBX does not target VACV protein synthesis. The action of CBX on VACV replication would, therefore, occur post protein production.

**Figure 3:**
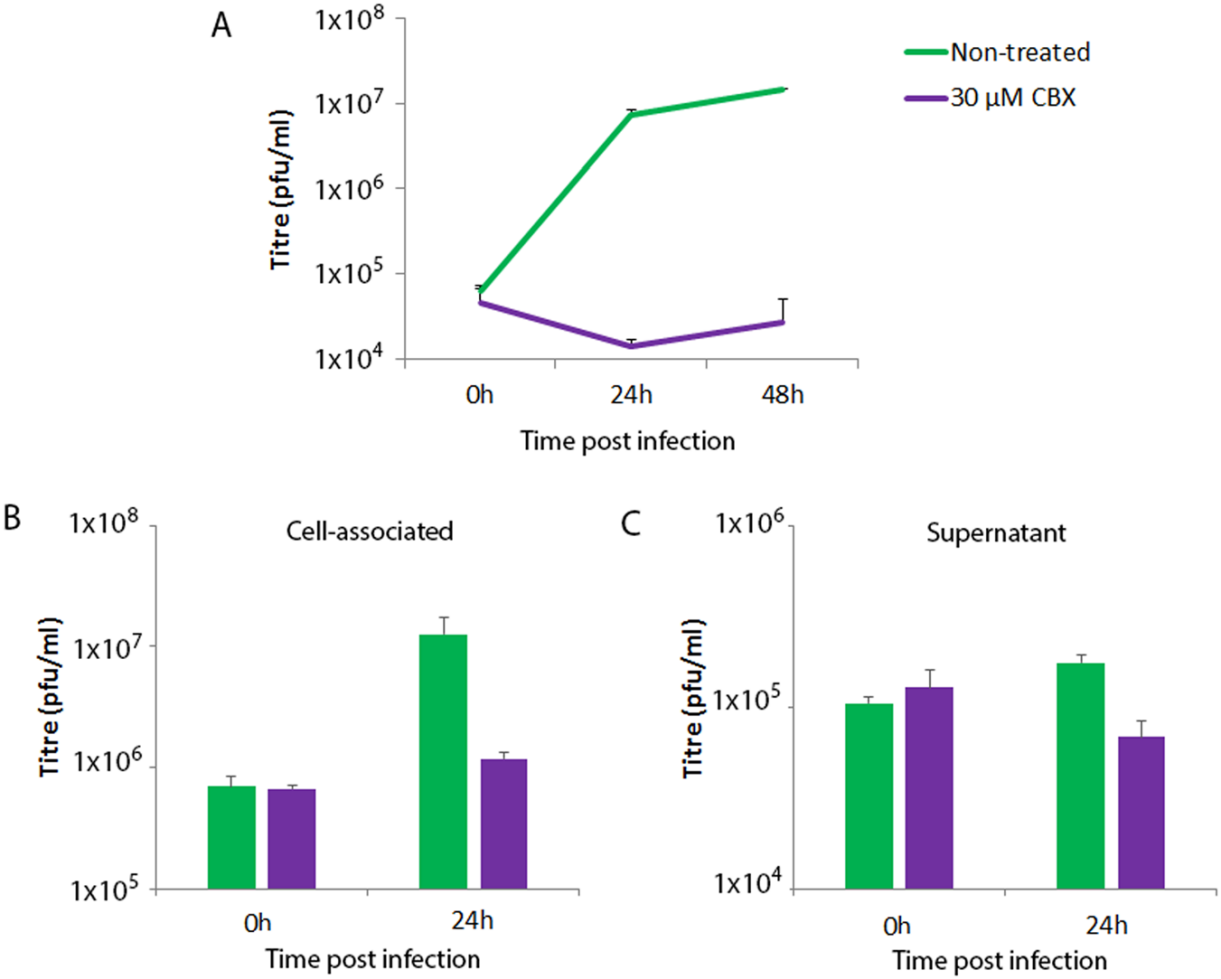
Carbenoxolone depletes virion formation. (**A**) Multi-step growth curve. HaCaT cells were mock-treated or treated with 30 µM CBX and infected with an MOI of 0.1 of VACV. At the indicated time points, total virus loads were determined by plaque assay in BS-C-1 cells. (**B**,**C**) Single-step growth curve. HaCaT cells were mock-treated or treated with 30 µM CBX and infected with an MOI of 5 of VACV. At the indicated time points, virus loads in the cell associated (**B**) and supernatant (**C**) fractions were determined by plaque assay in BS-C-1 cells. Experiments are representative of two independent replicates. Error bars = standard deviation.

To determine the stage (s) of viral replication affected by CBX, we performed single- and multi-step growth curves in HaCaT cells, followed by titration in BS-C-1 cells. In a multi-step growth curve, HaCaT cells were infected at a low MOI (0.1), allowing for multiple cycles of virus replication to occur. As expected, viral titres increased by over 2 log_10_ in untreated cells, however CBX treatment (30 µM) strongly inhibited virus titres, containing them to input levels (Fig. 3A), indicating a potent influence of the drug on viral replication. In a single-step growth curve, cells were infected at a high MOI (5) and supernatants and cell associated fractions collected separately 24 h p.i. VACV produces two main infectious particles, intracellular mature virus (IMV) and extracellular enveloped virus (EEV). The former represents the vast majority of virus progeny and is released upon cell lysis whereas the latter is a small fraction of the total virus progeny, released from approximately 8 h p.i. from the surface of infected cells and important for long range virus dissemination *in vitro* and *in vivo*^18^. Viral titres in the untreated control cells increased by 18 fold and 1.7 fold above the 0 h timepoint in IMV and EEV respectively after 24 h. However CBX treatment strongly impaired the production of both IMV, restricting the increase to only 1.8x that of the 0 h timepoint (Fig. 3B) and reduced the level of EEV to below that of the 0 h timepoint (Fig. 3C). Taken together, these data indicate that CBX is able to almost entirely block the formation of new virions.

We have established that CBX on its own has a strong inhibitory effect on VACV replication in human keratinocytes as shown by fluorescence and virus titration, and that this phenomenon is independent of both gap junctions and PP2A regulation. Virus protein production seems to be largely unaffected as levels of VACV-encoded proteins were not downregulated following CBX treatment, with the presence of the late protein A27 in CBX-treated cells suggesting that viral early protein expression and DNA replication also take place in the presence of the compound. These results point to CBX acting on one or more cellular processes involved in virion formation.

**Figure 4:**
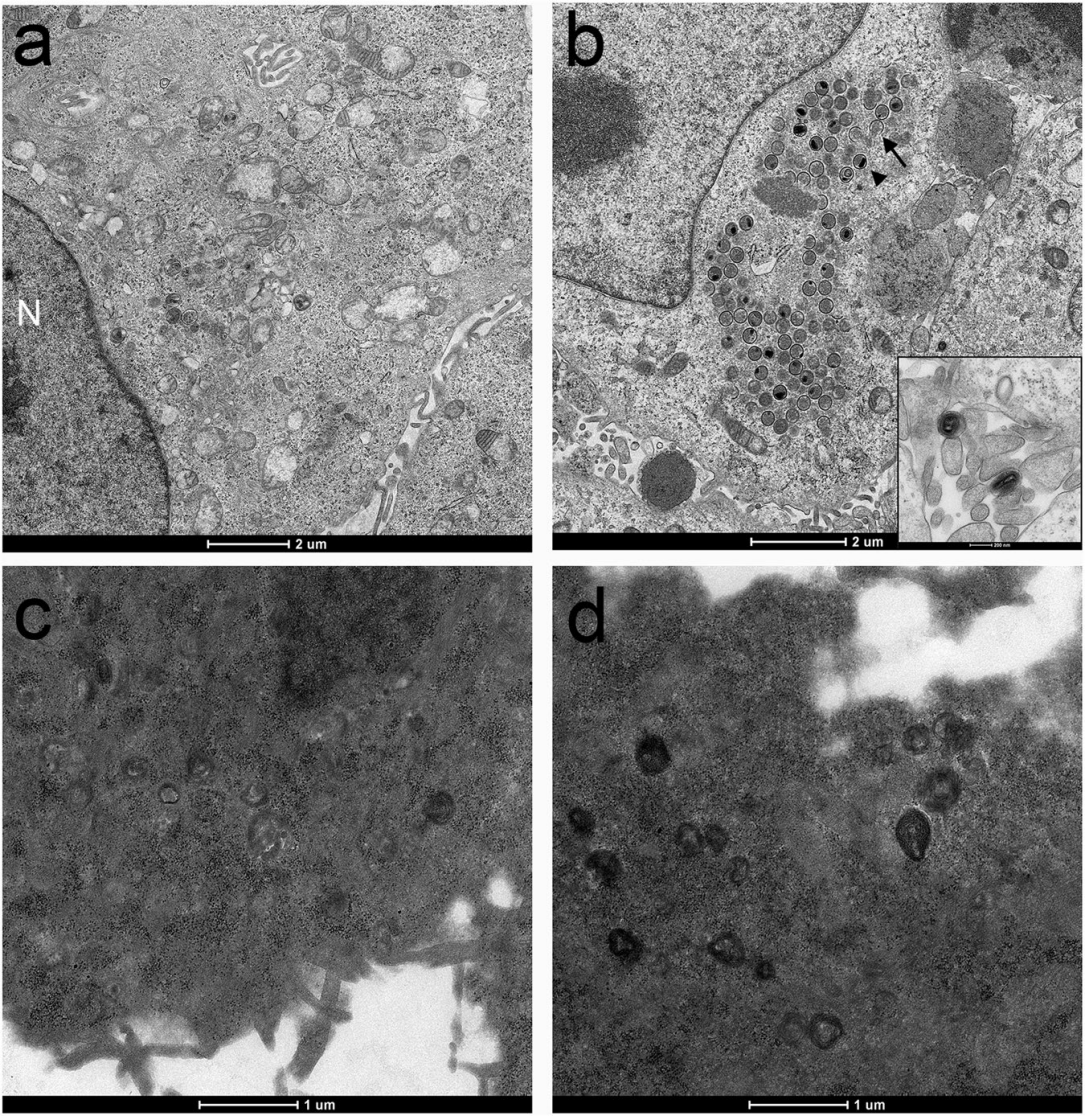
Electron microscopy of HaCaT cells, showing untreated and uninfected cells (**a**), cells infected with VACV (MOI = 5) (**b**), uninfected cells treated with 30 µM CBX (**c**), and cells treated with 30 µM CBX and infected with VACV (MOI = 5) (**d**). Panel A shows the cell nucleus (N) and cytoplasmic organelles. Panel B demonstrates the early stages of VACV assembly, with abundant crescents (arrow) and immature virions (arrowhead) within the viral factory. The insert shows two mature virions.

In order to investigate this further HaCaT cells were either mock infected or infected with VACV in the presence or absence of CBX (30 µM) and samples processed for ultrastructural study using transmission electron microscopy. We observed normal cellular ultrastructure in uninfected and untreated cells (Fig. 4a), and classic VACV virion morphogenesis in the VACV-infected, mock treated cells (Fig. 4b). In contrast the CBX-treated mock and infected cells exhibited widespread and severe destruction of normal cellular morphology with loss of ultrastructural detail such as intracellular membranous structures (Fig. 4c,d). These features are consistent with cellular necrosis. No virus particles, either normal or abnormal, were observed in the cytoplasm of CBX-treated cells (Fig. 4d). CBX treatment of HaCaT cells impairs the processes involved in VACV virion formation, most likely via impacts on the endomembrane system of the cell.

The cellular damage caused by CBX treatment evident at ultrastructural level was surprising given the absence of indicators of toxicity in HaCaT cells using the CellTiter-Blue assay. Other studies, albeit in different cell lines, have used CBX at much higher concentrations (up to 150 µM) than the 30 µM used in this study^19,20^. These results highlight the importance of multiple measures of cell viability.

This work indicates that careful analysis of the effects of CBX on cells should be included when using this compound in experimental work. Similarly, the impact of other potential antiviral compounds on the cellular ultrastructure should be studied in more detail. Such studies would not only aid the characterisation of antiviral functions but also the further development of potential new antiviral compounds and the understanding of complex cellular processes.

## Methods

### Cells and viruses

Immortalised human keratinocytes (HaCaT), murine neuroblastoma (N2a) and African green monkey kidney epithelial (BS-C-1) cells were grown in Dulbecco’s modified Eagle’s medium (DMEM) (Life Technologies) with 10% foetal bovine serum (Life Technologies), 50 IU/ml penicillin and 50 µg/ml streptomycin (Sigma). Cells were maintained at 37 °C in a 5% CO_2_ incubator. VACV strain Western Reserve (WR) and VACV-A5L-EGFP^13^ were a gift from Prof Geoffrey L. Smith (University of Cambridge). Sucrose purified IMV were used in these experiments.

### Fluorescence and cell viability experiments

HaCaT and N2a cells were seeded in 96-well plates at a density of 5 × 10^4^ cells per well and incubated at 37 °C for 24 h. Cells were either mock treated or treated with different concentrations of CBX for 1 h before infection and then throughout the experiment. Cells were infected in triplicate with VACV-A5L-EGFP (MOI = 0.1) for 1 h at 37 °C, inocula were removed and cells overlaid with 2.5% DMEM (DMEM containing 2.5% FBS (Life Technologies), 50 IU/ml penicillin and 50 µg/ml streptomycin (Sigma)). Fluorescence levels were measured at 0, 24 and 48 h p.i. using a Synergy HT Multi-Mode Microplate Reader (BioTek). Cell viability was measured at 48 h p.i. using CellTiter-Blue ® (Promega) according to manufacturer’s instructions.

### SDS-PAGE and immunoblotting

HaCaT cells were seeded at 5 × 10^5^ cells per well in a 12-well plate and incubated at 37 °C for 24 h. When required, cells were pre-treated with 30 µM CBX for 1 h ahead of virus infection and then throughout the experiment. Cells were infected with VACV WR at an MOI of 5 for 1 h at 37 °C. Inocula were removed and cells overlaid with 2.5% DMEM. Whole cell lysates were collected into lysis buffer^21^ at 0, 6 and 24 h p.i., separated by electrophoresis following quantification with the Pierce™ BCA Protein Assay Kit (Thermo Scientific) and either stained with SimplyBlue™ Safe Stain (Invitrogen) according to manufacturer’s instructions and visualised using the ChemiDoc™ XRS Imaging System (Bio-Rad) or transferred onto PVDF membranes (Merck). Blots were treated with Odyssey® blocking buffer (LI-COR Biosciences) before incubation with antibodies against PP2A (2038, Cell Signaling Technology), A27 (NR-627, BEI Resources) and actin (3700, Cell Signaling Technology). Secondary antibodies were goat anti-rabbit IgG (H + L) DyLight® 800 conjugate and goat anti-mouse IgG (H + L) DyLight® 680 conjugate, both from Cell Signalling Technology. Blots were scanned using the Odyssey® Imaging System (LI-COR Biosciences).

### Single and multi-step growth curves

HaCaT cells were either treated with 30 µM CBX or mock treated for 1 h before infection with VACV WR for 1 h at 37 °C, followed by removal of the inocula and addition of 2.5% DMEM. Cells pre-treated with CBX were infected in the presence of the compound and it was also added to the respective overlays.

For the multi-step growth curves, an MOI of 0.1 of VACV WR was used. Cells were harvested at 0, 24 and 48 h p.i. by scraping them into the medium. Samples were submitted to three freeze/thaw cycles, sonicated and titrated in BS-C-1 cells. For the single-step growth curves, cells were infected with VACV WR at an MOI of 5. At 0 and 24 h p.i. the supernatants were collected, centrifuged at low speed to remove any cell debris and incubated with L1 antibody (NR-417, BEI Resources) to neutralise IMV particles prior to titration in BS-C-1 cells. Cells were scraped into medium, collected by low speed centrifugation and submitted to three freeze/thaw cycles ahead of sonication and titration in BS-C-1 cells.

### Virus titration

Samples were serially diluted in 2.5% DMEM. Dilutions were then inoculated onto confluent monolayers of BS-C-1 cells in 6-well plates. Each dilution was inoculated in duplicate. Infection took place for 1 h at 37 °C and was followed by the removal of the inocula and the addition of 2 ml of 1.5% carboxymethylcellulose (Sigma) in 2.5% DMEM. Cells were then incubated for 2 days at 37 °C. The overlay was removed, cells were briefly washed in PBS and fixed/stained with 0.1% (w/v) crystal violet (Sigma) in 15% ethanol. Plates were rinsed with water, air-dried and the number of plaques counted.

### Electron Microscopy

HaCaT cells were seeded onto 13 mm Thermanox™ coverslips (ThermoFischer Scientific) and incubated for 48 h at 37 °C. Some cells were treated with 30 µM CBX for 1 h prior to virus infection and then throughout the protocol until the fixation step. Cells were either mock infected or infected with VACV WR (MOI = 5) for 1 h at 37 °C, inocula were removed, cells overlaid with 2.5% DMEM and incubated for 16 h at 37 °C. Cells were fixed in 2% glutaraldehyde followed by post-fixation in 1% aqueous osmium tetroxide solution (Agar Scientific) and dehydration in graded ethanols. Coverslips were transferred to polythene cups and washed with propylene oxide ahead of infiltration with 1:1 mix of propylene oxide and Agar 100 epoxy resin (Agar Scientific) and then 100% Agar 100 epoxy resin. After overnight polymerisation at 60 °C, 80 µm thin sections were cut, collected onto copper grids and grid stained used Leica EM AC20 before being imaged at 100 kV in a FEI Tecnai 12 TEM with a TVIPS F214 digital camera.
